# E-Cadherin Expression Varies Depending on the Location within the Primary Tumor and Is Higher in Colorectal Cancer with Lymphoid Follicles

**DOI:** 10.3390/cancers15123260

**Published:** 2023-06-20

**Authors:** Adam R. Markowski, Konstancja Ustymowicz, Anna J. Markowska, Wiktoria Romańczyk, Katarzyna Guzińska-Ustymowicz

**Affiliations:** 1 Department of Internal Medicine and Gastroenterology, Polish Red Cross Memorial Municipal Hospital, 79 Henryka Sienkiewicza Street, 15-003 Bialystok, Poland; 2 Medical University of Warsaw, 61 Żwirki i Wigury Street, 02-091 Warsaw, Poland; 3 Department of General Pathomorphology, Medical University of Bialystok, 13 Jerzego Waszyngtona Street, 15-269 Bialystok, Polandkguzinska74@gmail.com (K.G.-U.)

**Keywords:** cancer advancement, intratumoral heterogeneity, tumor microenvironment, tumor budding, biomarkers, personalized medicine

## Abstract

**Simple Summary:**

Different distributions of E-cadherin expression within colorectal cancer were observed; the highest percentage of positive E-cadherin expression was found in the invasive front and in the tumor center. Additionally, the different cellular distribution of E-cadherin expression was noticed; weak membranous E-cadherin expression was the highest in the invasive front and in the budding sites, but a strong membranous pattern was most frequent in the tumor center. Various distributions of E-cadherin expression depending on cancer progression were also found; E-cadherin expression in node-positive patients was lower in the tumor center and in the tumor invasive front, whereas, in patients with distant metastases, the expression of E-cadherin was lower in the budding sites. In tumors with lower TNM stages, with lymphoid follicles, and with some lower tumor budding parameters, E-cadherin expression was higher. E-cadherin expression was revealed to be lower at the tumor center in younger individuals, at the budding sites in men, and at the surrounding lymph nodes in rectal tumors.

**Abstract:**

Reliable indicators of cancer advancement have actively been sought recently. The detection of colorectal cancer progression markers is essential in improving diagnostic and therapeutic protocols. The aim of the study was to investigate the profile of E-cadherin expression in colorectal cancer tissue depending on the TNM staging and its correlation with several clinical and histopathological features. The study included 55 colorectal cancer patients admitted to the surgical ward for elective surgery. Tissue samples were obtained from resected specimens. Different distributions of E-cadherin expression within tumors were observed; the highest percentage of positive E-cadherin expression was found in the invasive front and in the tumor center. Additionally, the different cellular distribution of E-cadherin expression was noticed; weak membranous E-cadherin expression was the highest in the invasive front and in the budding sites, but a strong membranous pattern was most frequent in the tumor center. Various distributions of E-cadherin expression depending on cancer progression were also found; E-cadherin expression in node-positive patients was lower in the tumor center and in the tumor invasive front, whereas, in patients with distant metastases, the expression of E-Cadherin was lower in the budding sites. In patients with higher TNM stages, E-cadherin expression was lower within the tumor (in the budding sites, tumor center, and invasive front). In tumors with lymphoid follicles, E-cadherin expression was higher in all localizations within the primary tumor. E-cadherin expression in the tumor center was also lower in tumors with some higher tumor budding parameters (areas of poorly differentiated components and poorly differentiated clusters). E-cadherin expression was found to be lower at the tumor center in younger individuals, at the budding sites in men, and at the surrounding lymph nodes in rectal tumors. Low E-cadherin expression appears to be a reliable indicator of higher cancer staging and progression. When assessing the advancement of cancer, apart from the TNM classification, it is beneficial to also consider the expression of E-cadherin. High tumor budding, the poverty of lymphoid follicles, and low E-cadherin expression analyzed simultaneously may contribute to a reliable assessment of colorectal cancer staging. These three histopathological features complement each other, and their investigation, together with conventional tumor staging and grading, may be very helpful in predicting the prognosis of colorectal cancer patients and qualifying them for the best treatment. The role of E-cadherin in the diagnosis and treatment of colorectal cancer, as a part of a personalized medicine strategy, still requires comprehensive, prospective clinical evaluations to precisely target the optimal therapies for the right patients at the right time.

## 1. Introduction

Colorectal cancer is a potentially deadly and simultaneously preventable multifactorial disease with etiology encompassing genetic factors, environmental exposures, diet, and inflammatory conditions of the bowel. Several factors increasing the risk of CRC can be modified to reduce the risk of developing the disease, including smoking, high consumption of red meat, low intake of fruits and vegetables, excessive alcohol drinking, low physical activity, and obesity [1]. CRC is still the second-most common cause of cancer death in the United States, its incidence decline slowed from 3–4% annually during the 2000s to 1% annually during the previous decade, and its mortality decreased by 2% annually at the time overall but increased by 0.5–3% annually in individuals younger than 50 years [2]. There is also a shift to left-sided tumors, with the proportion of rectal cancer increasing from 27% to 31% in 2019 [2].

The presence of hypoxia, chemoattractants, the stiffness of the extracellular matrix, and the lack of nutrients prompt cancer cells to invade and migrate [3]. The epithelial-mesenchymal transition determines the plasticity of tumor cells, enabling them to switch from a non-motile epithelial phenotype to a motile mesenchymal state and endowing them with many malignant features, such as increased invasiveness, as well as resistance to aging, apoptosis, and chemotherapy [3]. Thanks to this process, cancer cells are able to leave the primary tumor, infiltrate the basement membrane, migrate through the extracellular matrix, penetrate endothelial cells to reach blood vessels, and thus spread throughout the body, creating secondary sites where they can multiply, resulting in organ failure. The E-cadherin protein determines the adhesion of cells in the tissue, and the reduction in its expression usually indicates the beginning of an epithelial–mesenchymal transition.

Conventional prognostic parameters for CRC include tumor staging and grading, metastases, and, additionally, lymphatic, perineural, and venous invasion. However, one study also assessed E-cadherin expression depending on these markers, and it turned out that most CRC patients had tumors with low (60%) E-cadherin expression, which was significantly associated with a higher T stage, the presence of lymph node metastasis, and TNM staging [4]. In another recent study, the immunohistochemical staining, protein expression, and mRNA level of E-cadherin were lower in tumors than in normal tissues, and the expression levels of E-cadherin were significantly associated with the pathological classification, lymph node, distal metastasis, and TNM stage [5]. In addition, low E-cadherin expression in CRC tissue was significantly associated with poorer 5-year survival rates and turned out to be an independent prognostic factor for CRC patients [5].

The development of individualized treatment strategies for colorectal cancer is crucial. Precise prognostic and predictive biomarkers are useful factors in classifying patients for the optimal type of treatment. For this purpose, extensive scientific research aimed at thoroughly understanding the nature of CRC is being carried out to find better ways to prevent, detect, and treat it [6]. However, even recent studies confirm a cure rate of only 65% for colorectal cancer and a still-high (35%) probability of treatment failure, even despite surgery and adjuvant treatment [7]. A poor outcome is related to the advanced local invasion and development of distant metastases. Early diagnosis is difficult, as the initial symptoms of CRC are often nonspecific and occur in many other conditions. Therefore, to improve the poor prognosis of patients with CRC, we require the detection of reliable CRC progression markers and better stratification analyses. Understanding all aspects of cancer progression will aid in the development of effective cancer detection and therapy.

It seems that most colorectal cancers probably start off as benign polyps. The early detection and removal of polyps can effectively prevent the occurrence of CRC, and screening options are colonoscopy, computed tomographic colonography, stool-based tests, and biomarkers (genomic, epigenetic, transcriptomic, proteomic, metabolomic) [8]. Although surgery remains the definitive treatment modality, new chemotherapeutic agents will likely improve outcomes for CRC patients, and the advancement of new targeted therapies may bring further benefits. The application of artificial intelligence algorithms for detection and decision support for healthcare professionals may be helpful [1]. Since postoperative recurrence and metastasis are the main reasons for the low survival rate, it is imperative to find markers that can predict prognosis in CRC patients. The objective of this study was to analyze the E-cadherin expression in colorectal cancer tissue depending on the TNM staging (tumor, nodes, metastasis) and, additionally, some pathomorphological markers, such as tumor budding and lymphoid follicles.

## 2. Materials and Methods

### 2.1. Patients

The present retrospective study included 55 consecutive patients who were admitted to the surgical ward for elective colorectal surgery. A diagnosis of CRC was established using colonoscopy and the histopathological assessment of biopsied specimens or based on a CT scan. All patients were briefed on every aspect of their participation in the study, gave their cognizant consent, and confirmed their decision with a dated signature.

The study was conducted in accordance with the Declaration of Helsinki and was accepted by the Ethical Committee for Human Studies of the Medical University of Bialystok, approval no. R-I-002/228/2018.

### 2.2. CRC Tissue Samples

Tumor tissue samples (tumor and regional lymph nodes) were obtained from resected specimens. The dissected tissues were fixed with 10% buffered formalin. The histopathologic assessment was performed on formalin-fixed and paraffin-embedded sections of tissue specimens. Conventional staining with hematoxylin and eosin of primary tumor serial sections was selected for typical histological analysis. The pathologic examination using a standardized reporting template was accomplished on the resected specimens, and tumor staging was performed according to the American Joint Committee on Cancer criteria, version 8 guidelines [8]. CRC was classified according to the TNM staging (tumor, nodes, metastasis).

### 2.3. Tumor Budding Parameters

Tumor budding parameters located peritumorally and intratumorally were assessed as described previously [9].

Tumor budding foci (TBF) were defined as single isolated cancer cells or clusters of fewer than five cells located in the stroma or at the invasive margin of the tumor. The number of TBFs was counted within the field of the densest budding of 0.785 mm^2^ at 20× magnification and evaluated on a three-point scale: Low (TBF-1): 0–4 buds, intermediate (TBF-2): 5–9 buds, and high (TBF-3): ≥10 buds.

Poorly differentiated clusters (PDC) were defined as clusters comprising ≥five cancer cells. The number of PDC was rated on a three-point scale: Low (PDC-1): 0–4 clusters, moderate (PDC-2): 5–9 clusters, and severe (PDC-3): ≥10 clusters.

Areas of poorly differentiated components (POR) were defined as regions where cancer had no glandular formation. The lowest magnification of the objective lens for which the poorly differentiated component filled the field of vision was regarded as the extent of the poorly differentiated component of CRC. POR was estimated on a three-point scale: Low (POR-1): areas occupied the greater part of the tumor, moderate (POR-2): areas did not occupy the greater part of the tumor, and severe (POR-3): areas did not occupy the field with a ×40 objective lens.

### 2.4. Lymphoid Follicles

Lymphoid follicles are small collections of B cells, T cells, and supporting cells. In our study, the presence of lymphatic follicles was assessed by the conventional staining with hematoxylin and eosin of tissue sections at 40× magnification.

### 2.5. E-Cadherin Expression

**Figure 1:**
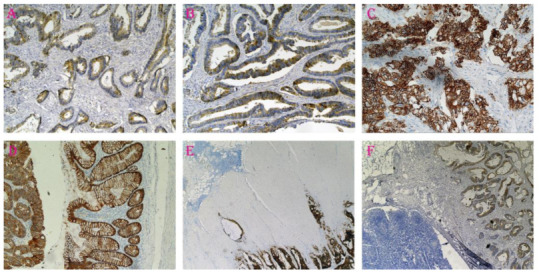
E-cadherin expression in colorectal cancer tissue resected from different patients. (**A**): ECD-1, weak membranous pattern, cytoplasmic distribution, ×20 magnification. (**B**): ECD-2, moderate membranous pattern, decreased cytoplasmic expression, ×20 magnification. (**C**): ECD-3, intense, strong membranous pattern of staining, ×20 magnification. (**D**): On the right side of the figure, a normal colonic mucosa with a positive expression of E-cadherin, and on the left, strong E-cadherin expression in cancer cells, ×100 magnification. (**E**,**F**): The lymphatic follicle in the front of the tumor invasion, ×40 magnification.

In brief, formalin-fixed, paraffin-embedded tissue specimens were cut on a microtome into 5 μm sections, which were then deparaffinized in xylene and hydrated in alcohol. To expose the antigen, the slides were heated in a microwave oven for 15 min in citric acid buffer (pH = 6.0). The activity of endogenous peroxidase was blocked by incubating the sections in 0.5% hydrogen peroxide in methanol. Next, the samples were incubated with monoclonal antibodies specific for E-cadherin (NCL-ECad, Novocastra Laboratories Ltd.; dilution 1:50, for 60 min); the reaction was performed with the ABC technique using a Novostain Super ABC Universal Kit (NCL-ABCm, Novocastra Laboratories Ltd. Newcastle upon Tyne, United Kingdom). Protein expression was observed at random using 10 fields of view. The immunohistochemical membranous staining intensity of E-cadherin (ECD) was graded in a semiquantitative fashion according to a four-point scale as follows: ECD-0: absent (no expression); ECD-1: weak membranous pattern, cytoplasmic distribution; ECD-2: moderate membranous pattern, decreased cytoplasmic expression; and ECD-3: intense, strong membranous pattern of staining (Figure 1).

### 2.6. Statistics

Statistical analysis was performed with Statistica 13.3 (TIBCO Software Inc., Palo Alto, CA, USA). The Mann–Whitney U test was used to evaluate the differences between two independent groups, the Kruskal Wallis test (with post hoc analysis) was used for three or more independent groups, and the Friedman test (with post hoc analysis) was used for three or more dependent groups. Correlations between groups were analyzed using the Spearman rank test and Spearman correlation coefficient (r). Statistical significance was assumed if a *p* value was less than 0.05.

## 3. Results

### 3.1. Characteristics of the Population

Of the 55 patients, 49% (n = 27) were female (Table 1). All patients were Caucasian. The mean age of CRC patients at diagnosis was 67 years (range 43–89). The largest group consisted of patients aged 60–69 years (32.73%) and 70–79 years (38.18%), and the smallest group had those aged 80 years and above (9.09%). In total, 41.82% of CRCs were located in the rectum. The depth of invasion was assessed as grade T3 in the vast majority (94.55%). Most patients had no evidence of distant metastases (89.09%), but lymph node involvement was observed in nearly half of the patients (47.27%). The number of patients in each group according to TNM classification was as follows: TNM-I 5.45%, TNM-II 41.82%, TNM-III 41.82%, and TNM-IV 10.91%. Perineural invasion was found in a small percentage of cases (7.27%), but the lymphovascular invasion was observed in a majority of cases (63.64%). Low-grade tumor budding foci (TBF-1) were visible in the vast majority (92.73%). The frequency distribution of the remaining tumor budding parameters was more variable, but in these cases, a low grade was also the most common: PDC-1 in 61.82% and POR-1 in 65.45% of patients. Lymphoid follicles (LF) were found in 45.45% of patients (Table 1).

### 3.2. E-Cadherin Protein Expression

Positive E-cadherin labeling was revealed in the majority of cases (Table 1). The E-cadherin expression pattern was determined in four different locations: in tumor budding sites (BS), in the invasive frontal region of the tumor (IF), in the tumor center (TC), and in regional lymph nodes (LN).

The highest percentage of positive E-cadherin expression was found in the invasive front (98.18%) and in the tumor center (98.18%); it was much smaller (76.37%) in the budding sites (ECD-IF vs. ECD-BS, *p* < 0.05; ECD-TC vs. ECD-BS, *p* < 0.05) and the smallest (43.64%) in the regional lymph nodes (ECD-IF vs. ECD-LN, *p* < 0.05; ECD-TC vs. ECD-LN, *p* < 0.05) (Figure S1 in Supplementary Files). Weak membranous expression of E-cadherin staining (ECD-1) was most commonly found in the invasive front (32.73%) and in the budding sites (32.73%), but a strong membranous pattern of staining (ECD-3) was most common in the tumor center (52.73%); it was much smaller in the regional lymph nodes (34.55%) and the smallest both in the budding sites (29.09%) and the invasive front (29.09%) (Figure S2 in Supplementary Files). Concerning the tumor center, the distribution of E-cadherin expression was the most interesting and progressively variable; 16.36% of CRCs presented weak membranous staining of E-cadherin (ECD-1), 29.09% presented moderate membranous staining (ECD-2), and 52.73% presented intense membranous staining (ECD-3) (Figure S1 in Supplementary Files). On the other hand, in tumor budding sites, a relatively uniform distribution was observed; 32.73% of CRCs presented weak membranous staining of E-cadherin (ECD-1), 14.54% presented a moderate pattern, and 29.09% presented intense membranous staining (ECD-3) (Figure S1 in Supplementary Files). Different distributions were observed in the invasive front (35.29%, 27.46%, and 1.96%, respectively) and in regional lymph nodes (5.88%, 37.26%, and 52.94%, respectively) (Figure S1 in Supplementary Files). The greatest differences were observed in the lack of E-Cadherin expression (Figure S2 in Supplementary Files).

In all CRC patients, intense membranous E-cadherin expression (ECD-3) was unequally distributed in different locations. It was the highest in the tumor center (52.73%), lower in lymph nodes (34.55%), and the lowest in the budding sites (29.09%) and in the invasive front (29.09%). E-cadherin expression in the budding sites was highly variable and showed no correlation with E-cadherin expression in any location. However, different relationships were found in the remaining sites. E-cadherin expression in the tumor center positively (and very strong) correlated with E-cadherin expression at the invasive front (r = 0.72; *p* < 0.0001) and negatively (strong) correlated with E-cadherin expression at the lymph nodes (r = −0.42; *p* = 0.0014). Additionally, E-cadherin expression in the invasive front negatively (strong) correlated with E-cadherin expression at the lymph nodes (r = −0.56; *p* < 0.0001).

**Figure 2:**
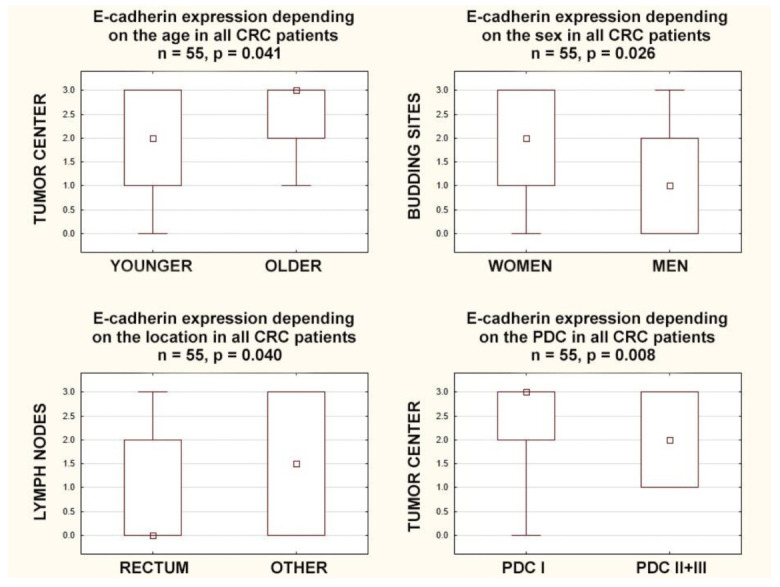
Box plots representing the association of membranous E-Cadherin expression in different locations with the age, gender, tumor location, and poorly differentiated clusters in CRC patients. The small square shows the median, the large rectangles demonstrate the 25–75% confidence interval, and the whiskers represent the minimum and maximum values.

Some age dependence was observed, as E-cadherin expression in the tumor center was lower in younger individuals, those below 70 years of age (*p* = 0.041). In turn, E-cadherin expression in budding sites positively correlated with the sex of the patients (r = 0.30; *p* = 0.024); in women, E-cadherin expression in the budding sites was higher (*p* = 0.026). Additionally, in rectal tumors, E-cadherin expression was lower in the surrounding lymph nodes compared to all other locations together (*p* = 0.040) (Figure 2).

### 3.3. Lymphoid Follicles

**Figure 3:**
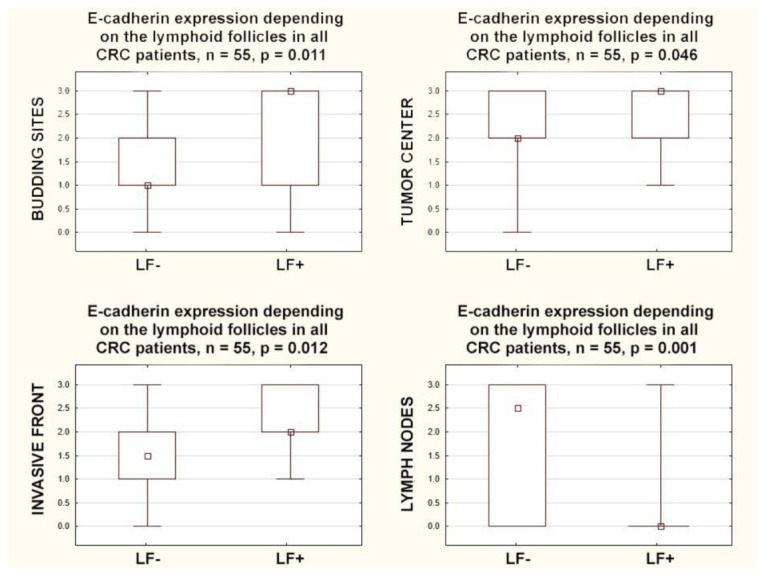
Box plots representing the association of membranous E-Cadherin expression in different locations with the presence of lymphoid follicles in CRC patients. The small square shows the median, the large rectangles demonstrate the 25–75% confidence interval, and the whiskers represent the minimum and maximum values.

E-cadherin expression within the primary tumor, in all localizations, positively correlated with the presence of lymphoid follicles: in the budding sites (r = 0.35; *p* = 0.009), in the tumor center (r = 0.27; *p* = 0.044), and in the invasive front (r = 0.34; *p* = 0.010). A completely different relationship was observed for the expression of E-cadherin in regional lymph nodes; a negative correlation with the presence of lymphoid follicles was found (r = −0.47 *p* = 0.0003). Furthermore, E-cadherin expression in all compartments within the primary tumor was also higher in CRCs with lymphoid follicles: in the budding sites (*p* = 0.011), in the tumor center (*p* = 0.046), and in the invasive front (*p* = 0.012). However, an inverse relationship was found in the lymph nodes because E-cadherin expression was lower in CRCs with lymphoid follicles (*p* = 0.001) (Figure 3).

### 3.4. Budding

E-cadherin expression in the tumor center and in the invasive front negatively correlated with poorly differentiated clusters (r = −0.40; *p* = 0.003. r = −0.28; *p* = 0.038, respectively), and E-cadherin expression in the tumor center negatively correlated with areas of poorly differentiated components (r = −0.41; *p* = 0.002). Furthermore, E-cadherin expression in the tumor center was higher in CRCs with lower areas of poorly differentiated components (POR-1) than in those with higher ones (POR-2+3) (*p* = 0.005), and in the tumor center, it was higher in CRCs with lower poorly differentiated clusters (PDC-1) than in those with higher ones (PDC-2+3) (*p* = 0.008) (Figure 2).

### 3.5. E-Cadherin Expression According to TNM Classification

**Figure 4:**
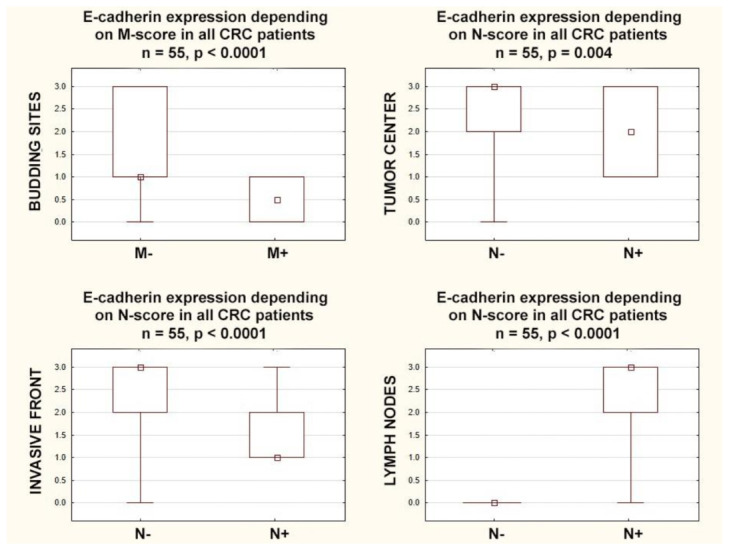
Box plots representing the association of membranous E-Cadherin expression according to N and M categories in CRC patients. The small square shows the median, the large rectangles demonstrate the 25–75% confidence interval, and the whiskers represent the minimum and maximum values.

E-cadherin expression in the lymph nodes positively correlated with lymph nodes involvement (r = 0.90; *p* < 0.0001), but E-cadherin expression in the tumor center and in the invasive front negatively correlated with lymph nodes involvement (r = −0.39; *p* = 0.003. r = −0.55; *p* < 0.0001, respectively). E-cadherin expression in the tumor center and in the invasive front was higher in node-negative CRCs (*p* = 0.004 and *p* < 0.0001, respectively), but E-cadherin expression in the lymph nodes was lower in node-negative CRCs (*p* < 0.0001). E-cadherin expression in the budding sites negatively correlated with distant metastases (r = −0.30; *p* = 0.024) and was higher in CRCs with distant metastases (*p* = 0.027) (Figure 4).

Similar relationships were not found in the analysis of the T category, but the above-described correlations for the N and M categories resulted in further dependencies; TNM classification was negatively correlated with E-cadherin expression in the budding sites (r = −0.29; *p* = 0.032), tumor center (r = −0.39; *p* = 0.003), and invasive front (r = −0.51; *p* = 0.0001), while it was positively correlated with E-cadherin expression in the lymph nodes (r = 0.66; *p* = 0.0001).

**Figure 5:**
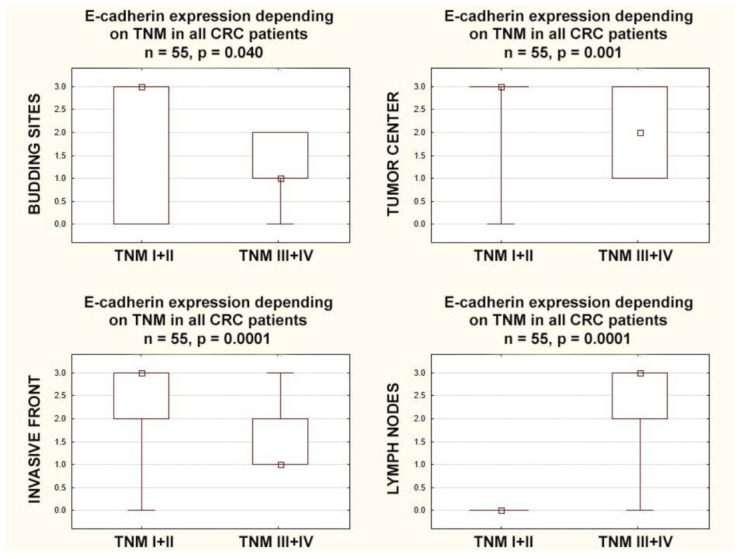
Box plots representing the association of membranous E-Cadherin expression according to different stages of TNM classification in CRC patients. The small square shows the median, the large rectangles demonstrate the 25–75% confidence interval, and the whiskers represent the minimum and maximum values.

In patients with higher stages (TNM-III+IV), E-cadherin expression was lower in the budding sites (*p* = 0.040), in the tumor center (*p* = 0.001), and in the invasive front (*p* = 0.0001). Additionally in patients with higher stages (TNM-III+IV), E-cadherin expression in the lymph nodes was (inversely) higher than in the lower stages (TNM-I+II) (*p* = 0.0001) (Figure 5).

## 4. Discussion

The luminal surface of the gastrointestinal tract is covered by a single layer of polarized, epithelial cells, which, thanks to being one of the fastest regenerating tissues in the body, form a robust, physical barrier that protects against numerous intestinal microbes or chemical and physical insults. Epithelial colonic cells undergo rapid and continuous self-renewal from the base of the crypts, where multipotent stem cells constantly divide, giving rise to mature cells. When they undergo apoptosis and are shed into the lumen, neighboring cells reform tight junctions to maintain cell polarization, cell-to-cell communication, a highly organized tissue structure, stabilization of the epithelial barrier, cell survival, and differentiation [10].

Adherent junctions use E-cadherin interactions to bind epithelial cells to their neighbors. E-cadherin consists of the extracellular, transmembrane, and intracellular domains, forming specialized structures called adherens junctions. These are also involved in cytoskeleton organization, intracellular signaling, and transcription regulation. The downregulation of E-cadherin in the cell membrane results in a loss of cell–cell and cell–extracellular matrix adhesion, inducing malignant phenotypes in normal cells and cancer progression [11]. Epithelial–mesenchymal transition (EMT) is a process during which epithelial cells acquire a less differentiated mesenchymal phenotype and behavior. This is because adherence junctions, apical tight junctions, and basolateral hemidesmosomes are disassembled and tightly packed epithelial cells lose attachment to neighboring cells and apical-basal polarity, form a loosely organized tissue with reduced intercellular adhesion, a gain of motility, migratory properties, and invasive ability, and have an increased resistance to apoptosis and an enhanced capacity of extracellular matrix production [12]. The hallmark of EMT is the downregulation of E-cadherin, and this process is regulated by a complex network of signaling pathways and transcription factors [13]. Since tumor buds can take on the properties of cells undergoing epithelial–mesenchymal transitions, it looks like they have a more invasive and migratory potential [14].

Our current study seems to confirm these assumptions. The cellular expression of E-cadherin showed changes depending on the location within the tumor and the distance from the tumor center. The highest percentage of positive E-cadherin expression was found in the tumor center and in the invasive front, while it was simultaneously clearly reduced in budding sites. Admittedly, a slightly different distribution of the particular E-Cadherin expression variants was found in the tumor center and in the invasive front, but these changes did not differ statistically in these two locations. One earlier study revealed a slightly different result, showing that the loss of E-cadherin was higher in the invasive front than in the tumor center [15]. However, our findings coincide with the result of another study [16] and suggest that cancer cells may change their phenotype before acquiring the ability to metastasize, which may result in a change in intracellular E-cadherin localization as well as its distribution within the tumor. We observed that the E-cadherin expression in the tumor center and in the invasive front was higher in node-negative patients but did not differ statistically despite the formation of distant metastases. In contrast, in patients with distant metastases, the expression of E-Cadherin in the budding sites was lower than that in patients without distant metastases, confirming suggestions that buds have a more invasive and migratory potential. Conversely, in the lymph nodes, we observed an extremely increased expression of E-cadherin in node-positive patients, as if, at this location, the cancer cells were recovering the phenotype from the primary tumor.

Additionally, the intracellular E-cadherin distribution in our patients showed a heterogeneous pattern. A cytoplasmic localization prevailed in the invasive front and in the budding sites, but the membranous patterns of staining were most common in the primary tumor center; to our knowledge, this is the first observation of this kind. Other studies also quantified the profile of E-cadherin expression in CRC patients, albeit without assessing the distribution within the tumor; the results were inconclusive, as some studies showed an advantage in the cytoplasmic localization [17,18], and in others, the membranous distribution of E-cadherin was found to be superior [19]. The results of another recent study showed that E-cadherin was mainly localized in membranous and nuclear fractions in two colorectal cancer cell lines; this aberrant nuclear localization promoted colorectal tumor progression [20]. On the other hand, according to other authors, membranous E-cadherin expression had no associations with prognosis, whereas positive cytoplasmic E-cadherin staining was a predictor of a more ominous outcome [17].

The majority of the gut-associated lymphoid tissue in the colon is composed of isolated lymphoid follicles dispersed throughout the large intestines. These lymphoid follicles have a diameter of 0.1–0.7 mm and consist of a specialized epithelium that overlies a subepithelial copula containing numerous macrophages, dendritic cells, lymphocytes, and antigen-sampling cells [21]. In our study, E-cadherin expression in all locations inside the primary tumor (invasive front, tumor center, budding sites) was higher, and in regional lymph nodes, it was lower in CRCs with lymphoid follicles; this is the first observation of this kind and seems to broaden our understanding of CRC biology. Some authors have previously shown that lymphoid follicles have immune-mediated anti-tumor effects [21]. Others have suggested that lymphoid follicles in early colorectal tumors are signs of an early physical defense event against cancer cells [22]. In our recent study, we found that the presence of lymphoid follicles positively correlated with the density of some tumor-infiltrating immune cells (lymphocytes CD8 and tumor-associated neutrophils) in the tumor center and with the density of some tumor-infiltrating immune cells (lymphocytes CD8) in the invasive front [9]. Moreover, we found that a high lymphoid follicles density was observed in cases of low-advanced cancer according to the TNM stage; it appears that lymphoid follicles may reflect the host’s defense against cancer and be an indicator of a favorable prognosis [9].

In our patients, the lymph node category (N of TNM) negatively correlated with E-cadherin expression in the tumor center and in the invasive front but positively correlated (and particularly strongly) with E-cadherin in the regional lymph nodes. The metastasis category (M of TNM), in turn, negatively correlated with E-cadherin expression in budding sites. This fact was reflected in the results of the E-cadherin expression analysis, depending on the CRC stage and according to the TNM classification. In patients with a higher TNM stage, E-cadherin expression was lower in the tumor center and in the invasive front. Conversely, a different relationship was shown for the expression of E-cadherin in the regional lymph nodes, as it was lower in early CRC than in patients with advanced CRC. Other studies also showed that the reduced expression of E-cadherin in tumor tissue was linked with advanced-stage tumors and with a higher staging category T, N, and M [23,24,25,26,27].

Additionally, in the current study, E-cadherin expression in the tumor center and in the invasive front was negatively correlated with some tumor budding parameters. This fact agrees with the observations of another study indicating that the loss of E-cadherin was related to high tumor budding [15]. Further investigation showed that poorly differentiated clusters and tumor buds displayed less E-cadherin expression than tumor centers [28]. Moreover, the abnormal (cytoplasmic) staining pattern for E-cadherin was also more common in poorly differentiated clusters and tumor buds than in the tumor centers [28]. In our patients, E-cadherin expression in budding sites was, to some extent, dependent on the sex of the patients, and it was lower in men; to our knowledge, this is the first observation of this kind, and for now, it is difficult to say whether this phenomenon will find practical application. Other previous studies found that E-cadherin expression was sex-independent, but those studies did not specify in which parts of the tumor (tumor center, budding sites, invasive front) E-cadherin expression was determined [29,30].

In our study, E-cadherin expression was slightly lower in younger patients (*p* = 0.041), but only in the tumor center; such a phenomenon could favor the onset of colorectal cancer at an earlier age. In budding sites, the invasive front, and lymph nodes (*p* = 0.367, *p* = 0.235, *p* = 0.939, respectively), no such relationship was found. So far, the data on this point are ambiguous. In one study, membranous staining for E-cadherin was observed in 74.3% of colorectal cancers, E-cadherin expression evaluated semi-quantitatively was also associated with the age at diagnosis, and a loss of E-cadherin expression was more often demonstrated in younger (≤60 years) patients (47.4% vs. 17.6%) [31]. On the other hand, in another study, a loss of E-cadherin was less frequently detected in younger patients (<55 years) [32]. Probably, these dependencies are more complex and not yet fully explained. In other studies, lower E-cadherin expression was associated with a larger tumor size [31], a more advanced CRC stage at diagnosis, a tendency toward lymph node involvement [33], lymphovascular and perineural invasion [15], a worse clinical response to treatment [34], more frequent recurrences, shorter 5-year disease-free survival [35], shorter 10-year survival [36], and a worse response to neoadjuvant therapy [37]. E-cadherin expression in the primary tumor (budding sites, tumor center, invasive front) was independent of cancer localization (rectum, sigmoid, colon, cecum), although lymph node E-cadherin expression was lower in rectal tumors than in tumors located in other parts of the large intestine (*p* = 0.040). Other authors have shown that CRC localization has no significant association with E-cadherin expression [29]. However, not all research results are consistent on this issue as well. In some studies, E-cadherin expression was not associated with the tumor grade (at various stages of advancement) [29,34,38], distant metastasis [18,19], lymph node involvement [39,40], or prediction of mortality [18,38].

To date, there are only a few studies simultaneously evaluating the E-cadherin expression and other tumor markers in CRCs, and of course, they were not definitely clear-cut. In some CRC patients, no correlation was found between the E-cadherin expression and tumor budding [41,42]. In one study, the expression of both membranous and cytoplasmic E-cadherin was lower in tumor budding sites than in the tumor center [43]. In two other studies, the loss of E-cadherin was associated with high tumor budding [15,44]. The results of our study are in agreement with these two reports.

Now, we add the new important observation, complementing previous findings, that E-Cadherin expression (in the tumor center and in the invasive front) is higher in low-advanced CRCs and lower in high-advanced CRCs. A recent meta-analysis including 9591 colorectal cancer patients seems to strongly support our findings, as it was found that low E-cadherin expression was significantly associated with the more advanced neoplastic disease: a shorter overall survival and disease-free survival, a higher risk of low differentiation, a high risk of distant metastasis, a high risk of vascular invasion, a higher risk of lymph node metastasis, a high risk of lymphatic invasion, and a high risk of deep infiltration [45].

The current study has its limitations. It was a retrospective study conducted at one university hospital. We did not evaluate the molecular mechanism of different E-cadherin expression patterns in miscellaneous tumor regions, nor the potential role of tumor-infiltrating immune cells in altering E-cadherin expression. Future investigations of this aspect may provide interesting new data and insights.

## 5. Conclusions

Low E-cadherin expression appears to be a reliable indicator of higher cancer staging and progression. We propose that, in the assessment of the advancement of colorectal cancer apart from the TNM classification, E-cadherin expression should also be taken into account. It seems that the combined analysis of some pathomorphological markers, independent of TNM staging, assessed within a colorectal cancer tissue, such as high tumor budding, scanty lymphocyte infiltration, the poverty of lymphoid follicles, and low E-cadherin expression in the tumor center and in the invasive front, may contribute to a reliable assessment of CRC staging. These biomarkers complement each other, and their evaluation may prove very helpful in predicting prognosis and qualifying CRC patients for the best treatment.

The TNM staging provides a strong and accurate prognosis for patients with early and advanced colorectal cancer. However, there are inaccuracies in the prognostication of the intermediate stages of CRC, suggesting a need to improve the scales used and a complex and not fully understood interaction of ultimately unidentified factors. The recognition of them can help precisely distinguish patients who have intermediate-stage colorectal cancer according to the TNM classification but who are at a high risk of aggressive progression, metastasis, and recurrence. However, the role of E-cadherin in the diagnosis and treatment of colorectal cancer, as a personalized medicine strategy, still requires comprehensive prospective clinical evaluations to precisely target the most optimal therapies for the right patients at the best time.

## Figures and Tables

**Table 1:** Clinical and pathological characteristics of the study group (n = 55).

**Gender**
Male, n = 27 (49.09%)
Female, n = 28 (50.91%)
**Age**
<60, n = 11 (20.00%)
60–69, n = 18 (32.73%)
70–79, n = 21 (38.18%)
≥80, n = 5 (9.09%)
**Primary tumor location**
Rectum, n = 23 (41.82%)
Colon, n = 32 (58.18%)
**Histologic type**
Adenocarcinoma, n = 54 (98.18%)
Mucinous adenocarcinoma, n = 1 (1.82%)
**T stage, Depth of tumor invasion**
T1, submucosa, n = 0
T2, muscularis propria, n = 3 (5.45%)
T3, subserosa, n = 52 (94.55%)
T4, serosa or other organs, n = 0
**N stage, Lymph node metastases**
N0, absent, n = 29 (52.73%)
N1, present, n = 26 (47.27%)
**M stage, Distant metastases**
M0 absent, n = 49 (89.09%)
M1 present, n = 6 (10.91%)
**TNM stage**
TNM-I, n = 3 (5.45%)
TNM-II, n = 23 (41.82%)
TNM-III, n = 23 (41.82%)
TNM-IV, n = 6 (10.91%)
**Lymphovascular invasion (LVI)**
LVI-0, absent, n = 20 (36.36%)
LVI-1, present, n = 35 (63.64%)
**Perineural invasion (PNI)**
PNI-0, absent, n = 51 (92.73%)
PNI-1, present, n = 4 (7.27%)
**E-cadherin expression pattern in the tumor center (ECD-TC)**
ECD-TC-0, n = 1 (1.82%)
ECD-TC-1, n = 9 (16.36%)
ECD-TC-2, n = 16 (29.09%)
ECD-TC-3, n = 29 (52.73%)
**E-cadherin expression pattern in the invasive front (ECD-IF)**
ECD-IF-0, n = 1 (1.82%)
ECD-IF-1, n = 18 (32.73%)
ECD-IF-2, n = 20 (36.36%)
ECD-IF-3, n = 16 (29.09%)
**E-cadherin expression pattern in tumor budding sites (ECD-BS)**
ECD-BS-0, n = 13 (23.63%)
ECD-BS-1, n = 18 (32.73%)
ECD-BS-2, n = 8 (14.55%)
ECD-BS-3, n = 16 (29.09%)
**E-cadherin expression pattern in regional lymph nodes (ECD-LN)**
ECD-LN-0, n = 31 (56.36%)
ECD-LN-1, n = 2 (3.64%)
ECD-LN-2, n = 3 (5.45%)
ECD-LN-3, n = 19 (34.55%)
**Tumor budding foci in colorectal cancer tissue (TBF)**
TBF-1, n = 51 (92.73%)
TBF-2, n = 3 (5.45%)
TBF-3, n = 1 (1.82%)
**Poorly differentiated clusters in colorectal cancer tissue (PDC)**
PDC-1, n = 34 (61.82%)
PDC-2, n = 14 (25.45%)
PDC-3, n = 7 (12.73%)
**Areas of poorly differentiated components in colorectal cancer tissue (POR)**
POR-1, n = 36 (65.45%)
POR-2, n = 12 (21.82%)
POR-3, n = 7 (12.73%)

## Data Availability

The data that support the findings of our study are available from the corresponding authors upon reasonable request and with the permission of the respondents.

## Supplementary Materials

The following supporting information can be downloaded at: https://www.mdpi.com/article/10.3390/cancers15123260/s1, Figure S1. Histograms representing the intensity of membranous E-Cadherin expression distribution in four different locations in CRC patients. Figure S2. Histograms representing the localization of four membranous E-Cadherin expression patterns in CRC patients.
