# Supplementary figures and images for: E-Cadherin Expression Varies Depending on the Location within the Primary Tumor and Is Higher in Colorectal Cancer with Lymphoid Follicles
Source: Cancers (Basel). 2023 Jun 20;15(12):3260. doi: 10.3390/cancers15123260 (PMC10296849; doi:10.3390/cancers15123260)

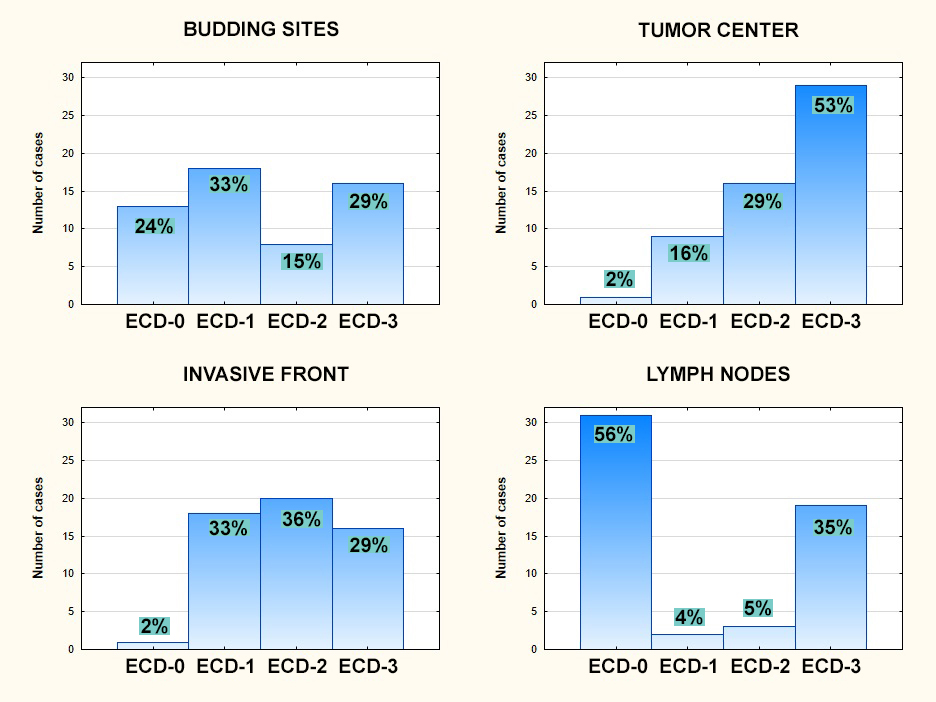

Supplement: Supplementary file 1 [file cancers-15-03260-s001.zip › supplementary files/Figure S1-Markowski E-cadherin Histogram 1 A-D poprawiony.jpg]

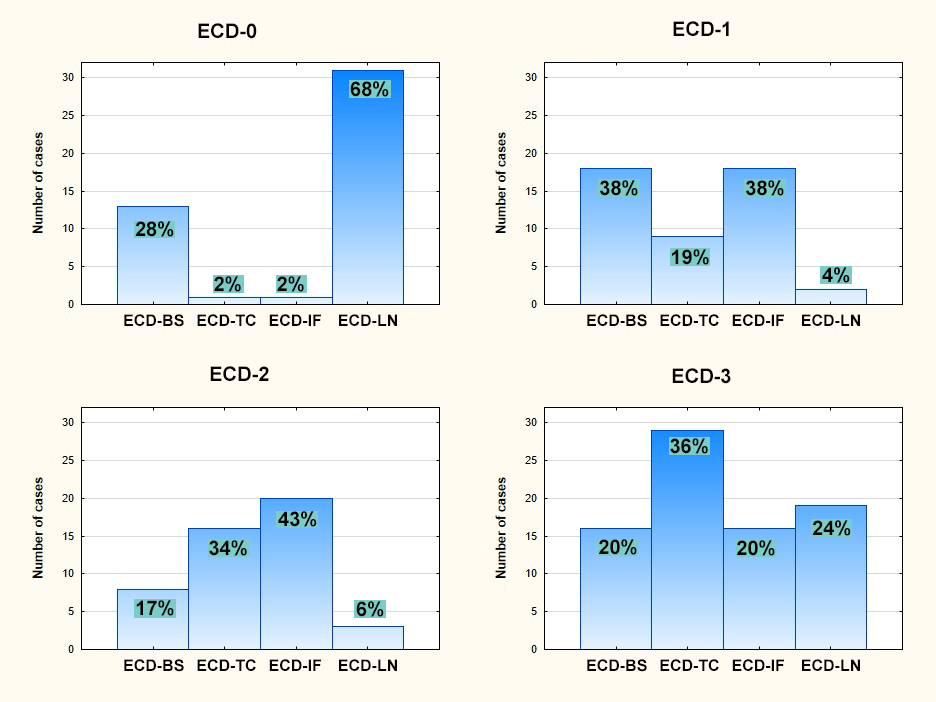

Supplement: Supplementary file 1 [file cancers-15-03260-s001.zip › supplementary files/Figure S2-Markowski E-cadherin Histogram 2 A-D poprawiony.jpg]
